# Enhancing the 1-Aminocyclopropane-1-Carboxylate Metabolic Rate of *Pseudomonas* sp. UW4 Intensifies Chemotactic Rhizocompetence

**DOI:** 10.3390/microorganisms8010071

**Published:** 2020-01-02

**Authors:** Xiyang Gao, Tao Li, Wenliang Liu, Yan Zhang, Di Shang, Yuqian Gao, Yuancheng Qi, Liyou Qiu

**Affiliations:** Ministry of Agriculture and Rural Affairs, Key Laboratory of Enzyme Engineering of Agricultural Microbiology, Henan Agricultural University, College of Life Sciences, Zhengzhou 450002, China

**Keywords:** 1-aminocyclopropane-1-carboxylic acid deaminase, chemoattractant, chemotaxis, metabolic rate, plant growth promotion, *Pseudomonas* sp., rhizocompetence, rhizosphere colonization

## Abstract

1-aminocyclopropane-1-carboxylic acid (ACC) is a strong metabolism-dependent chemoattractant for the plant beneficial rhizobacterium *Pseudomonas* sp. UW4. It is unknown whether enhancing the metabolic rate of ACC can intensify the chemotaxis activity towards ACC and rhizocompetence. In this study, we selected four promoters to transcribe the UW4 ACC deaminase (AcdS) gene in the UW4 Δ*AcdS* mutant. PA is the UW4 *AcdS* gene promoter, PB20, PB10 and PB1 are synthetic promoters. The order of the *AcdS* gene expression level and AcdS activity of the four strains harboring the promoters were PB20 > PA > PB10 > PB1. Interestingly, the AcdS activity of the four strains and their parent strain UW4 was significantly positively correlated with their chemotactic activity towards ACC, rhizosphere colonization, roots elongation and dry weight promotion. The results released that enhancing the AcdS activity of PGPRenable them to achieve strong chemotactic responses to ACC, rhizocompetence and plant growth promotion.

## 1. Introduction

Plant roots secrete up to 30% of their fixed carbon to closely interact with rhizosphere bacteria [1]. Applying plant growth-promoting rhizobacteria (PGPR) bioinoculants not only reduces the amount of chemical fertilizer required by 20–30% and pesticides required by more than 30% but also increases the yield of crops by 10–80%. However, the PGPR perform in field conditions is often very impoverished compared to that in laboratory conditions. An approach to solve this problem is using higher concentrations of microbial preparations [2], but this approach frequently fails due to their high cost [3]. An alternative to address this issue is improving the carrier formulation of the inoculants to lengthen the shelf life of the inoculants and enhance the survival of PGPR in the field [4].

The approaches above, however, have neglected the mechanism by which PGPR promote plant growth. It has been confirmed that the mechanism is that PGPR recognize root exudates as chemoattractants, move towards the rhizosphere through chemotaxis, utilize root exudates for proliferation and colonize the rhizosphere niche, subsequently promoting plant growth directly or indirectly by secreting a variety of metabolites [5]. Chemotaxis is the first crucial step and intensifies the rhizocompetence of PGPR in colonizing the rhizosphere [3]. Two issues should be addressed to enhance the chemotaxisrhizocompetence of PGPR. The first is determining which compound in root exudates is the key chemoattractant for PGPR chemotaxis to the rhizosphere, and the second is determining how to improve the chemotactic response activity of PGPR to the key chemoattractant [6].

In addition to the common trait of PGPR colonizing the rhizosphere, another important trait is that most PGPR produce 1-aminocyclopropane-1-carboxylate (ACC) deaminase (ACC deaminase, AcdS). AcdS catalyzes the cleavage of ACC to ammonia and α-ketobutyrate, enabling PGPR to utilize ACC as a carbon and nitrogen source. ACC is a nonprotein amino acid that is a precursor to the synthesis of ethylene in plants and is abundant in seeds and root exudates, especially under stress conditions [7]. The AcdS produced by PGPR degrades the ACC and reduces the synthesis of ethylene in plants, relieving the ethylene inhibition of plant growth and development and enhancing the stress resistance of plants. Plants continuously secrete ACC to the rhizosphere to maintain dynamic equilibrium of the ACC in the rhizosphere, thus providing a continuous supply of nutrients for PGPR [8]. Therefore, nonplant pathogens possessing AcdS always exhibit plant-promoting effects and have a close reciprocal relationship with plants [9].

There have been several reports implying that ACC may be a key chemoattractant for PGPR chemotaxis to the rhizosphere. The rhizosphere colonization of *AcdS* mutants is significantly reduced compared to that of the wild-type strain [10]. Rhizosphere colonization, legume nodulation, and survival in the soil of PGPR without AcdS enzymatic activity are increased by introducing the *AcdS* gene [11,12]. In addition, most of the bacteria inhabiting the rhizosphere or endophytic bacteria under stress conditions are AcdS-producing bacteria but not producers of siderophores, indole-3-acetic acid and organic acids [13]. Even under control conditions, more PGPR possess *AcdS* among the rhizobacteria than produce siderophores [14]. Siderophore production is suggested to be the one unique trait for PGPR rhizocompetence [3]. Therefore, we propose that the ACC secreted by the roots should be the key chemoattractant for AcdS-producing PGPR in rhizosphere colonization.

To verify the hypothesis, we found that *Pseudomonas* sp. UW4, a typical PGPR strain, was chemotactic towards ACC and that ACC was a strong and metabolism-dependent chemoattractant for the bacterium. The rhizosphere colonization by UW4 mutants with chemotaxis or AcdS activity defects was dramatically reduced, while the colonization by UW4 was significantly increased or decreased with increased or decreased ACC secretion hosts, respectively, suggesting that ACC must be the key chemoattractant for the AcdS-producing PGPR in rhizosphere colonization. Therefore, enhancing the chemotactic response to ACC might intensify the chemotactic rhizocompetence of PGPR for rhizosphere colonization [5].

Several factors affect the chemotactic response of bacteria to a chemoattractant. A higher affinity of the methyl-accepting chemotaxis protein (MCP) to the corresponding ligand mediated a stronger chemotactic response [15]. Highly abundant MCPs also mediated a strong chemotactic response [16]. When *E. coli* was exposed to two opposing but equally potent gradients of attractants, the chemotactic responses of the cells were mediated by the highly abundant MCP over the other attractant chemoreceptor [17]. The methylation modification and clustering of chemoreceptors [18] and the metabolic rate for the metabolism-dependent chemoattractants are probably the additional factors that affect the bacterial chemotactic response activity. For example, the chemotactic response by *Azospirillum brasilense* was strongest in cells freshly harvested but was lost after several hours of starvation in buffer [19]. High expression levels of the UW4 *AcdS* led to high ACC metabolic rates, fitness and plant growth promotion by *A. brasilense* Cd [11]; however, it is unknown whether the high expression of the UW4 *AcdS* led to an increased chemotactic response of the strains to ACC.

In the present study, we used bacterial promoters of different strengths to express the *AcdS* gene in the UW4 *AcdS* deletion mutant and investigated the effect of ACC metabolic rate on the UW4 chemotactic response activity towards ACC, rhizosphere colonization and root elongation.

## 2. Materials and Methods

### 2.1. Microbes and Plants

*Pseudomonas* sp. UW4 and its *AcdS* deletion mutant UW4 Δ*AcdS* were kindly provided by Professor Bernard R. Glick, University of Waterloo (UW, Waterloo, ON, Canada). Wheat (*Triticum aestivum* L.) variety Aikang 58 seeds were obtained from the National Engineering Research Centre for Wheat (NERCW, Zhengzhou, China).

### 2.2. Bacterial Promoters

Three bacterial promoters, PB20, PB10 and PB1, rank-ordered by expression level from high-to-low, and UW4 *AcdS* gene promoter PA were selected to express the *Pseudomonas* sp. UW4 *AcdS* gene in UW4 Δ*AcdS*. The promoters PB20, PB10 and PB1 were from a synthetic promoter library for fine-tuning constitutive bacterial transcription [20], and their sequences are listed in Table S1. The UW4 *AcdS* gene promoter PA is an ACC-inducible promoter with a very high sensitivity [21].

### 2.3. Construction of Expression Vectors

The promoters and the downstream gene *AcdS* were cloned by PCR using *Pseudomonas* sp. UW4 genome DNA as template and four primer pairs, PB20-F+PB-R, PB10-F+PB-R, PB1-F+PB-R, PA-F+PA-R (Table S1). The PCR amplification conditions were 94 °C for 5 min; 30 cycles at 94 °C for 30 s, 66 °C for 30 s, and 72 °C for 125 s; and elongation at 72 °C for 10 min. The PCR products were ligated to the multiple cloning sites of the shuttle expression plasmid pBBR1MCS2 (confers kanamycin resistance) [22] generating four *AcdS* expression vectors. The expression vectors were maintained in *E. coli* DH5α.

### 2.4. Three-Parent Mating and Transconjugant Identification

The *AcdS* expression vectors were introduced into *Pseudomonas* sp. UW4 Δ*AcdS* using three-parent mating [23]. *E. coli* DH5α carrying the *AcdS* expression vector was used as a donor cell, *E. coli* DH5α harboring helper plasmid pRK2013 [23,24] was used as a helper cell, and UW4 Δ*AcdS* was used as a recipient cell. The transconjugants were isolated in Luria broth (LB) plates supplemented with kanamycin at 100 μg/mL and confirmed by vector extraction and identification and PCR. The vectors from the transconjugants were extracted using a SanPrep Column Plasmid DNA Miniprep Kit (Sangon Biotech Co., Ltd., Shanghai, China) following the manufacturer’s protocol and then double-digested by restriction enzymes *Bam*HI and *Hind*III. Two fragments harbored only in the *AcdS* expression vectors were amplified using colony PCR. One of the fragments corresponding to *AcdS* was amplified with primer pair AcdS-F+AcdS-R (Table S1), resulting in a PCR product 200 bp in size that corresponded to the *AcdS* located in the introduced plasmid, whereas a PCR product of 1636 bp corresponded to the UW4 Δ*AcdS* genome because the *AcdS* gene in the UW4 Δ*AcdS* genome included a tetracycline resistance gene expression cassette 1436 bp long. The second fragment, corresponding to the kanamycin resistance gene, was amplified with primer pair Kan-F+Kna-R (Table S1). All the PCR conditions were the same as above.

### 2.5. Growth Curves

*Pseudomonas* sp. UW4 strains were grown on LB medium or ACC glycerol salt medium, which is an alternative glycerol salt medium [25] containing 3 mM ACC instead of (NH_4_)_2_SO_4_, and shaken on a rotating shaker (220 rpm) at 30 °C for 18 h or 56 h. Growth curves were monitored by turbidimetric measurements at 600 nm and at 1–4 h intervals.

### 2.6. Quantitative Real-Time Reverse Transcription PCR

The *AcdS* gene expression levels of the *Pseudomonas* sp. UW4 strains were determined by quantitative real-time reverse transcription PCR (qRT-PCR). The RNA of the UW4 strains was used as a template, and primer pairs *AcdS*-F+*AcdS*-R and gyrB-F+gyrB-R (Table S1) were used to amplify the *AcdS* gene and the reference gene *gyrB*, respectively [26]. The PCR conditions were 95 °C for 30 s and 40 cycles at 95 °C for 5 s and 60 °C for 30 s. The related gene expression level was calculated by the 2^−ΔΔ*C*T^ method [27].

### 2.7. AcdS Enzyme Activity Assay

*Pseudomonas* sp. UW4 strains were cultivated in LB medium containing 3 mM ACC at 30 °C for 10 h, and the bacterial cells were harvested by centrifugation at 5000× *g* and 4 °C for 5 min. The AcdS activities were determined by measuring the amount of α-ketobutyrate produced from the cleavage of ACC catalyzed by AcdS in the supernatant of the bacterial cell lysis [28,29]. The protein concentrations of the cell extracts were determined using the Bradford method [30]. One unit of AcdS activity was defined as the amount of enzyme required to release 1 μmol α-ketobutyrate per minute under 30 °C.

### 2.8. Swimming Plate Chemotaxis Assay

A swimming plate chemotaxis assay was conducted as described previously [5]. Briefly, the chemoattractants were added to semisolid medium (10% LB medium) plates to a final concentration of 0.3 mM. A cell suspension (2 μL) was gently pipettedinto the center of the plate and incubated at room temperature. The chemotactic activity of the strain towards the chemoattractant was expressed as the percentage of the swimming diameter of the strain relative to that of the wild-type strain *Pseudomonas* sp. UW4.

### 2.9. Axenic Tissue Culture Bottles Trial

Colonization of wheat roots by *Pseudomonas* sp. UW4 strains in axenic tissue culture bottles were assessed using the described method [5,31] with some modified protocol. Sterile wheat seeds were germinated in Petri plates containing distilled water at room temperature for 3 d, and then the germinated seeds with the same size were transplanted into 300 mL plant tissue-culture flasks (Meilun Biotech Co., Dalian, China) each containing 50 mL solid MS media. After incubating in a growth chamber at 25 °C with 80% humidity and 16h/8h light/dark regime for 7 d, each three seedlings were fixed in a foam board and placed in a flask containing 50 mL liquid MS media and incubated on a shaker at 40 rpm in the growth chamber. Each treatment had three replicates and experiments were performed three times. After 1 d of shock culturing 1 mL of bacterial suspension of UW4 strains (OD_600_ =1.0) was added and continuously cultured for 3 d to determine the amount of bacteria colonized in the wheat rhizosphere by using serial dilutions plates. The plate medium was LB agar having penicillin 100 μg/mL for the quantification of UW4, while LB agar having 100 μg/mL penicillin and tetracycline 15 μg/mL for the quantification of the other strains. UW4 has natural penicillin resistance, the tetracycline resistance of UW4 Δ*AcdS* originated from the resistance marker inserted in the genome *AcdS* gene. The number of colony forming units (CFUs) per gram of root fresh weight (FW) was obtained by subtracting the numbers of the bacteria (CFU from medium with penicillin) of the uninoculated roots (Control) from the numbers of the bacteria (CFU from medium with penicillin or tetracycline) of the inoculated roots.

### 2.10. Pot Trial

Colonization of wheat roots by *Pseudomonas* sp. UW4 strains in pots were conducted following the procedures described by Penrose and Glick [32] and Fischer et al. [33] with slight modifications. Sterile wheat seeds were incubated with 0.03 M MgSO_4_ (used as control) or bacterial suspensions in sterile 0.03 M MgSO_4_ (adjusted OD_600_ =1.0) at room temperature for 3 h, then filtered out the solution, the seeds were pregerminated in Petri plates containing distilled water at room temperature for 1 d to show white sprouts. Each seedling was transferred to a pot containing about 100 g matrix of vermiculite:peat (1:1) and kept in a growth chamber with the same conditions as above. Six seedlings were used for each treatment; experiments were repeated three times. Five mL of sterile water was poured into each pot every day but the corresponding volume of the bacterial suspension (OD_600_ =1.0) was used instead of sterile water except the control for the first two days. The plants were finally harvested on pot cultivation for two weeks. The fresh and dry weight of the shoot and root of the plants were determined. The bacterial population on the roots were measured by using the method same as above.

### 2.11. Statistical Analysis

The data generated from the various strains were analyzed by multiple comparison post hoc tests using Graphpad prism 6.01 software.

## 3. Results

### 3.1. Identification of the Pseudomonas sp. UW4 ΔAcdS Transconjugants with AcdS Expression Vectors Controlled by Different Promoters

The UW4 Δ*AcdS* transconjugants introduced the *AcdS* expression vectors were grown on the kanamycin medium and further identified by vector characterization and amplification of the two fragments contained in only the *AcdS* expression vectors. The *AcdS* expression vectors were all extracted from the transconjugants and double-digested by *Bam*HI and *Hind*III to generate the promoter-*AcdS* fragments, which were 1.3 kb or 1.7 kb in size (Figure S1). The two fragments corresponding to *AcdS* or the kanamycin resistance gene were both amplified from the transconjugants to yield products with sizes of 0.2 kb or 1.0 kb (Figure S2). The resulting four transconjugants were named UW4 Δ*AcdS+*PB20-*AcdS*, UW4 Δ*AcdS+*PB10-*AcdS*, UW4 Δ*AcdS+*PB1-*AcdS* and UW4 Δ*AcdS+*PA-*AcdS*.

### 3.2. The Growth Curves of Pseudomonas sp. UW4 Strains

The growth curves of the six UW4 strains were tested with LB medium. The growth rates and final cell densities were not different among the strains (Figure S3A). However, growing on ACC glycerol salt medium, UW4 reached the log phase earlier than the four transconjugated UW4 Δ*AcdS* strains and had a higher final cell density. The transconjugated UW4 Δ*AcdS* strains rank-ordered by the time they took to reach the log phase from fastest to slowest were UW4 Δ*AcdS+*PA-*AcdS*, UW4 Δ*AcdS+*PB20-*AcdS*, UW4 Δ*AcdS+*PB10-*AcdS* and UW4 Δ*AcdS+*PB1-*AcdS*; while the final cell density of UW4 Δ*AcdS+*PA-*AcdS* was higher than that of the other three strains, it did not differ among the other three strains. UW4 Δ*AcdS* did not grow on ACC glycerol salt medium (Figure S3B).

### 3.3. The AcdS Gene Expression Levels in Pseudomonas sp. UW4 Strains

**Figure 1:**
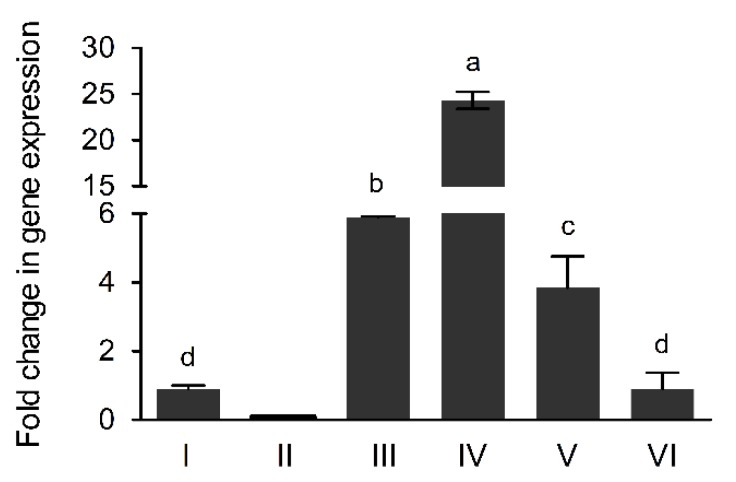
The *AcdS* gene expression levels in *Pseudomonas* sp. UW4 and derivative strains cultivated in LB medium plus ACC for 10 h. I, UW4; II, UW4 Δ*AcdS*; III, UW4 Δ*AcdS+*PA-*AcdS*; IV, UW4 Δ*AcdS+*PB20-*AcdS*, V, UW4 Δ*AcdS+*PB10-*AcdS*; VI, UW4 Δ*AcdS+*PB1-*AcdS*. All data are mean value of biological triplicates. Error bars represent standard errors. Data marked with different lowercase letters were statistically different at *p* < 0.05 based on the least significant difference (LSD) multiple comparison test.

The *AcdS* gene expression levels in UW4 strains were determined with LB medium containing ACC after 10 h of culturing. *AcdS* gene expression levels in the transconjugated and nontransconjugated strains decreased in the order UW4 Δ*AcdS+*PB20-*AcdS* > UW4 Δ*AcdS+*PA-*AcdS* > UW4 Δ*AcdS+*PB10-*AcdS* > UW4 Δ*AcdS+*PB1-*AcdS* and UW4 (Figure 1).

### 3.4. The AcdS Activities of Pseudomonas sp. UW4 Strains

**Figure 2:**
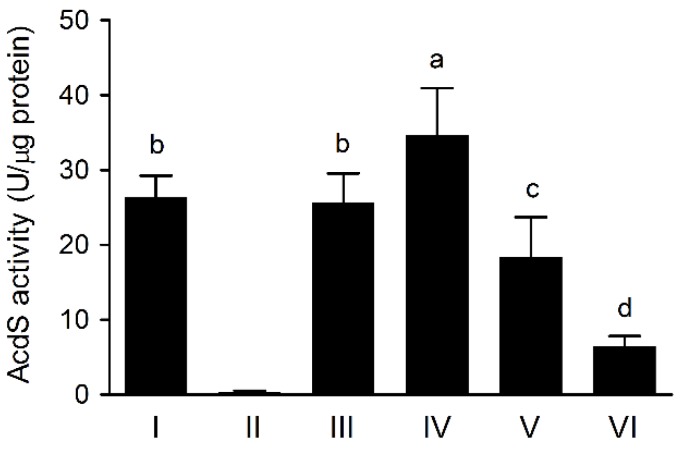
The AcdS activities of *Pseudomonas* sp. UW4 and derivative strains cultivated in LB medium plus ACC for 10 h. Note: Same as Figure 1.

The AcdS activities of UW4 strains were determined with LB medium containing ACC after 10 h of culturing. The AcdS activities of the transconjugated and nontransconjugated strains decreased in the order UW4 Δ*AcdS+*PB20-*AcdS* > UW4 Δ*AcdS+*PA-*AcdS* and UW4 > UW4 Δ*AcdS+*PB10-*AcdS* > UW4 Δ*AcdS+*PB1-*AcdS*, and the activity of UW4 Δ*AcdS* was unmeasurable (Figure 2). The AcdS activities of the three strains UW4 Δ*AcdS+*PA-*AcdS*, UW4 Δ*AcdS+*PB10-*AcdS* and UW4 Δ*AcdS+*PB1-*AcdS* were significantly positively correlated with their *AcdS* expression levels (*p* < 0.05). When the ratio of the enzyme activity to the gene expression level of UW4 is set at 1, the three strains UW4 Δ*AcdS+*PA-*AcdS*, UW4 Δ*AcdS+*PB10-*AcdS* and UW4 Δ*AcdS+*PB1-*AcdS* all exhibit ratios of approximately 1/6, and UW4 Δ*AcdS*+PB20-*AcdS* has a ratio of only approximately 1/20.

### 3.5. The Chemotaxis Response of Pseudomonas sp. UW4 Strains to Selected Chemoattractants

**Figure 3:**
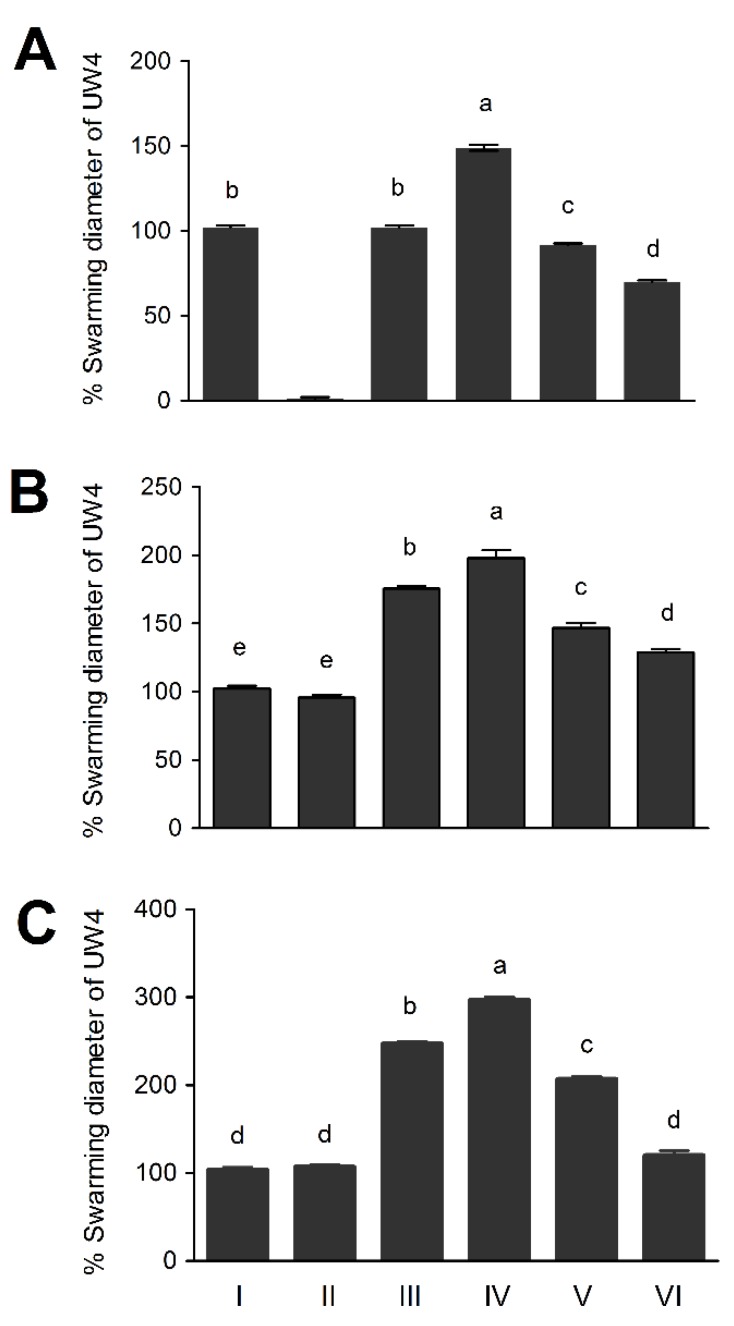
The chemotaxis response of *Pseudomonas* sp. UW4 and derivative strains to ACC (**A**), arginine (**B**) or succinic acid (**C**). Note: Same as Figure 1.

The chemotaxis response of UW4 strains to ACC was tested using a swimming plate chemotaxis assay. The chemotaxis response activity of the strains harboring the constitutive promoters to ACC, arginine or succinic acid decreased in the order UW4 Δ*AcdS+*PB20-*AcdS* > UW4 Δ*AcdS+*PB10-*AcdS* > UW4 Δ*AcdS+*PB1-*AcdS*. On the other hand, for the strains with the same inducible promoter located in plasmid or genome, the chemotaxis response activity of UW4 Δ*AcdS+*PA-*AcdS* to ACC was the same as that of UW4, while the chemotaxis response activities of UW4 Δ*AcdS+*PA-*AcdS* to either arginine or succinic acid were both higher than those of UW4 and UW4 Δ*AcdS*. UW4 Δ*AcdS* was not chemotactic towards ACC but was able to respond to arginine and succinic acid with similar intensities as UW4 (Figure 3). Furthermore, the ACC chemotaxis response activity of the five strains, not including UW4 Δ*AcdS*, was significantly positively correlated with their AcdS activities (*p* < 0.01). The chemotaxis response activities of the four strains, not including UW4 and UW4 Δ*AcdS*, towards arginine or succinic acid were also significantly positively correlated with their AcdS activities (*p* < 0.01).

### 3.6. Wheat Rhizosphere Colonization by Pseudomonas sp. UW4 Strains in Axenic Tissue Culture Bottles

**Figure 4:**
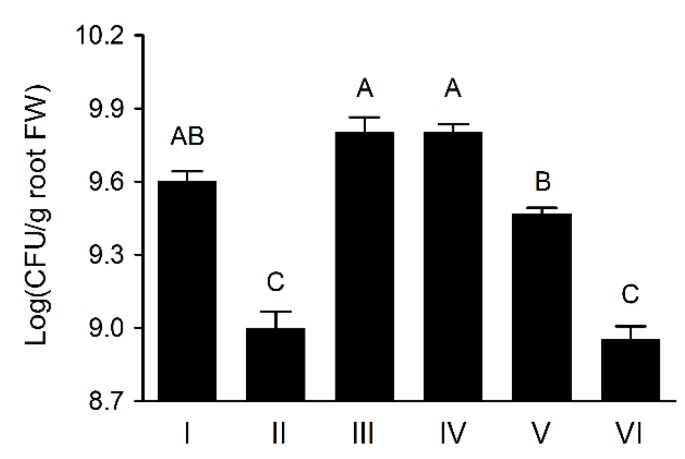
Colonization population in wheat rhizosphere by *Pseudomonas* sp. UW4 strains in axenic tissue culture bottles. Data marked with different uppercase letters were statistically different at *p*< 0.01 based on the least significant difference (LSD) multiple comparison test. Other notes: Same as Figure 1. The uppercase letters indicate the statistically different at *p* < 0.01.

The UW4 Δ*AcdS* cells were present on plant roots significantly less than that of UW4 after inoculation in axenic tissue culture bottles, agreeing with the previously reported observation [5,10], whereas the transconjugants of UW4 Δ*AcdS+*PA-*AcdS*, UW4 Δ*AcdS*+PB20-*AcdS* and UW4 Δ*AcdS*+PB10-*AcdS* recovered or increased the ability of UW4 Δ*AcdS* on wheat rhizosphere colonization, but the bacterial population of UW4 Δ*AcdS*+PB1-*AcdS* was much lower than that of UW4 for the duration of the experiment period (Figure 4). Interestingly, the bacterial populations of the strains in wheat rhizosphere, not including UW4 Δ*AcdS*, were significantly positive correlation with their AcdS enzymtic activities (*p* < 0.01).

### 3.7. Wheat Rhizosphere Colonization and Growth Promoting by Pseudomonas sp. UW4 Strains in Pot Trial

**Figure 5:**
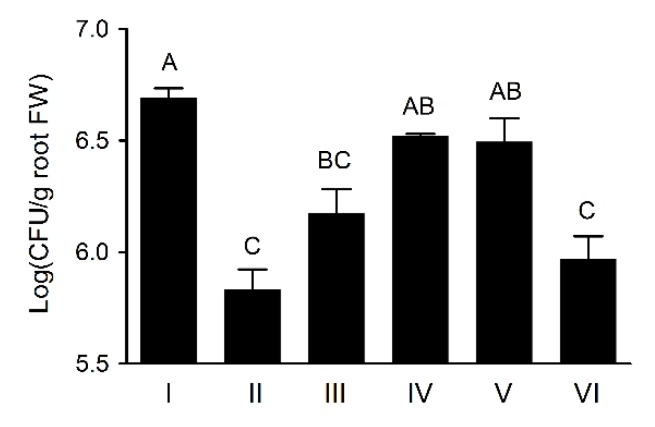
Colonizationpopulation in wheat rhizosphere by *Pseudomonas* sp. UW4 strains in Pot trial. Note: Same as Figure 4.

**Figure 6:**
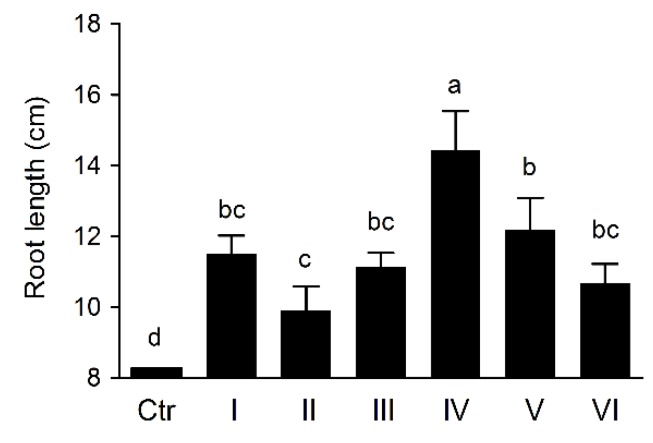
The effects of *Pseudomonas* sp.UW4 strains inoculation on wheat root length in Pot trial. Ctr, control with non-inoculation. Other notes: Same as Figure 1.

The bacterial populations colonized by UW4 strains in wheat rhizosphere tested in Pot trial were similar to that in axenic tissue culture bottles (Figure 5), and the bacterial cell accounts present on plant roots also significantly positive correlation with their AcdS enzymtic activities (*p* < 0.01), not including UW4 Δ*AcdS*. The furthermore, inoculating with UW4 strains was capable to significantly promote wheat roots elongation and dry weight in Pot trial (Figure 6 and Figure S4), and the AcdS activities of the strains, not including UW4 Δ*AcdS*, were significantly positive correlation with both roots elongation and dry weight (*p* < 0.01).

## 4. Discussion

In the present study, we created four *AcdS* expression vectortransconjugants of *Pseudomonas* sp. UW4 Δ*AcdS* controlled by four different promoters. The four promoters were rank-ordered by their strength in the following decreasing order: PB20, PA, PB10 and PB1. The expression vectors were derived from the pBBR1MCS2 plasmid. According to previous reports [34], the expression vector copy number in the transconjugants was approximately 20. High gene copy number and expression strength are double-edged swords to the host, because they can increase the expression level and enzyme activity of AcdS but also impose a greater metabolic load. Therefore, the transconjugant growth rate was obviously decreased in ACC glyceride minimal medium compared with LB medium. Meanwhile, the enzyme activities of the transconjugants did not increase in the same proportion as the gene expression level, possibly because of their mRNA lifespan and protein synthesis systems. For the relative weak promoters PA, PB10, and PB1, the ratio of the enzyme activity to the gene expression levelwas only approximately 1/6, while for the strong promoter PB20, the ratio was much lower only approximately 1/20.

The increasing amount of available information enables us to understand how metabolism affects metabolism-dependent chemotaxis [35]. There are two hypotheses to explain the mechanism by which metabolism influences thechemotaxis: one is the proton motive force hypothesis [36], and the other is the ATP phosphorylation hypothesis [35]. The proton motive force hypothesis suggests that cell metabolism should generate a change in the intracellular proton motive force; subsequently, aerotaxis and phototaxis are mediated by the change. The ATP phosphorylation hypothesis proposes that an increase in external metabolite concentration leads to ATP production, contributing to increased phosphorylation of CheY6 and, consequently, a higher probability to stop movement. However, the effect of the bacterial metabolic rate of the metabolisme-dependent chemoattractant on chemotaxis has not been reported.

In this study, we found that the promoters with higher expression levels produced higher activities of AcdS, higher ACC cell metabolism rates, and greater chemotaxis responses to ACC. The mechanism may be that the cell metabolizes the chemoattractant and further utilizes the metabolic products, relieving the chemoattractant metabolism feedback inhibition by the metabolic products and thus maintaining a high extracellular chemoattractant concentration gradient and a high proton potential or low ATP accumulation, leading to a strong, continual chemotactic response. Interestingly, the promoters with high expression levels not only produced high AcdS activity and a high chemotaxis response to ACC but also intensified chemotaxis responses to the other metabolisme-dependent chemoattractants such as amino acids and organic acids, possibly due to the high expression of AcdS disrupting the metabolic balance of the cells and accelerating the metabolic rate for other nutrients. Finally cells intensify the chemotaxis response to simultaneously varies metabolisme-dependent chemoattractants. We name such behavior the complementary product strategy.

Lots of studies confirmed a significant linear correlation between AcdS activity of PGPR and their root elongation promotion under axenic and pot conditions [37,38]; nevertheless, there have not been any reports about the correlation between AcdS activity of PGPR and their rhizosphere colonization. In several studies, AcdS activity of PGPR did not linearly correlate totheir number on plant roots [39,40], presumably due to rhizosphere colonization by PGPR being affected by not only the AcdS activity but also the chemotactic response and motility characteristics. Various strains have different chemotactic responses and motility characteristics, consequently making it difficult to establish the linear correlation between AcdS activity and rhizosphere colonization. In the present study, we found that AcdS activity of *Pseudomonas* sp. UW4 and the four *AcdS* expression transconjugants of UW4 Δ*AcdS* was linearly correlated not only with their ACC chemotactic response activity but also with their rhizosphere colonization, roots elongation and roots dry weight, indicating that enhancing the ACC metabolic rate of PGPR can intensify their chemotactic rhizocompetence for rhizosphere colonization.

The root exudation of certain classes of compounds, including sugars, amino acids, organic acids, phenolics, phytohormones, and other stress compounds, function as chemoattractants that PGPR detects and navigates toward the rhizosphere [41]. However, a large number of root exudates are also metabolic dependent chemoattractants for deleterious rhizobacteria [42] or nonrhizobacteria [43]. Consequently, PGPR must compete for the chemoattractants with other indigenous soil bacteria. Interestingly, most PGPRs contain AcdS, and can use ACC as the sole nitrogen source, giving them a significant advantage over indigenous bacteria. In previous studies, we found ACC is a key chemoattractant for AcdS-producing PGPRs in rhizosphere colonization [5]. The results of the present study further confirmed the aforementioned conclusions.

## 5. Conclusions

The conclusion from the resultswould suggest that the development of PGPR strains with high AcdS activity could reach the high chemotactic rhizocompetence, high rhizosphere colonization and high root growth promotion. To the best of our knowledge, this study is the first to demonstrate that enhancing the metabolic rate of metabolism-dependent chemoattractants can increase the bacterial chemotactic response to chemoattractants.

## Supplementary Materials

The following are available online at https://www.mdpi.com/2076-2607/8/1/71/s1, Figure S1. DNA electrophoresis of pBBR1MCS2 and derivative vectors extracted from *Pseudomonas* sp. UW4 Δ*AcdS* transconjugants and the *Bam*HI-*Hind*III-digested productions of the vectors. Figure S2. PCR products corresponding to the *AcdS* (A) and kanamycin resistance gene (B) amplified from *Pseudomonas* sp. UW4 Δ*AcdS* and transconjugants. Figure S3. The growth curves of *Pseudomonas* sp. UW4 and derivative strains growing on LB medium (A) and glycerol salt medium containing ACC as sole nitrogen source (B). Figure S4. The effects of *Pseudomonas* sp. UW4 strains inoculation on wheat root dry weight in Pot trial. Table S1. Primers used in this study.
