# Supplementary material for: Enhancing the 1-Aminocyclopropane-1-Carboxylate Metabolic Rate of Pseudomonas sp. UW4 Intensifies Chemotactic Rhizocompetence
Source: Microorganisms. 2020 Jan 2;8(1):71. doi: 10.3390/microorganisms8010071 (PMC7023479; doi:10.3390/microorganisms8010071)
Supplement: Supplementary file 1 [file microorganisms-08-00071-s001.zip › microorganisms-683725-supplementary.doc]

Supplementary Materials

**Figure S1.** DNA electrophoresis of pBBR1MCS2 and derivative vectors extracted from *Pseudomonas* sp. UW4 Δ*AcdS* transconjugants and the *Bam*HI-*Hind*III-digested productions of the vectors. M, 1kb Plus DNA Ladder; 1, pBBR1MCS2 undigested; 2, pBBR1MCS2 digested; 3, PB1+*AcdS*; 4, PB10+*AcdS*; 5, PB20+*AcdS*; 6, PA+*AcdS*; 7, UW4 Δ*AcdS*.

**Figure S2.** PCR products corresponding to the *AcdS* (A) and kanamycin resistance gene (B) amplified from *Pseudomonas* sp. UW4 Δ*AcdS* and transconjugants. M, 50 bp DNA Ladder (A) or 1kb Plus DNA Ladder (B); 1, UW4 (A) or pBBR1MCS2 (B); 2, PB1+*AcdS*; 3, PB10+*AcdS*; 4, PB20+*AcdS*; 5, PA+*AcdS*; 6, UW4 Δ*AcdS*.

**Figure S3.** The growth curves of Pseudomonas putida UW4 and derivative strains growing on LB medium (A) and glycerol salt medium containing ACC as sole nitrogen source (B). All data are mean value of biological triplicates. Error bars represent standard errors.

**Figure S4.** The effects of *Pseudomonas* sp. UW4 strains inoculation on wheat root dry weight in Pot trial. Ctr, control with non-inoculation. Other notes: Same as Fig. 4.

Table S1 Primers used in this study

| Primer | Sequence (5'–3')^a^ |
| --- | --- |
| PB20-F | CGGGATCC*AATACTTGACATATCACTGTGATTACATATAATATGCG*AAATCTGTAAGGCTAGCCAGGCTACACAGGGAATGAACCTGAATCGTTTTG *Bam*HI site shown by underlining |
| PB10-F | CGGGATCC*ACCTATTGACAATTAAAGGCTAAAATGCTATAATTCCAC*AAATCTGTAAGGCTAGCCAGGCTACACAGGGAATGAACCTGAATCGTTTTG *Bam*HI site shown by underlining |
| PB1-F | CGGGATCC*TCCCTTTGATATTGCATCCCGCGTATATAATATGTC*AAATCTGTAAGGCTAGCCAGGCTACACAGGGAATGAACCTGAATCGTTTTG *Bam*HI site shown by underlining |
| PB-R | CCAAGCTTGTCAATCACGTATTTGGGTAAC *Hind*III site shown by underlining |
| PA-F | CGGGATCCGGTTGAAACTCTGG *Bam*HI site shown by underlining |
| PA-R | CCAAGCTTTTTGACCCAGAC *Hind*III site shown by underlining |
| AcdS-F | ACTACATCGTGGTCTGCTCGGT |
| AcdS-R | CACATCCTCTTCAGTGATTTCG |
| Kna-F | GGGAGCGGCGATACCGTAAAGC |
| Kna-R | AAGTGCCACCTGGGATGAATGT |
| gyrB-F | CCTGTCGGAAGAGCTGGT |
| gyrB-R | ACCGTGGACGTAAGTCTGTT |

^a^ The sequence in italic is the synthetic promoter PB20, PB10 or PB1.
